# E3 ubiquitin ligase tripartite motif-containing 71 promotes the proliferation of non-small cell lung cancer through the inhibitor of kappaB-α/nuclear factor kappaB pathway

**DOI:** 10.18632/oncotarget.19075

**Published:** 2017-07-07

**Authors:** Hongjiu Ren, Yitong Xu, Qiongzi Wang, Jun Jiang, Wudumuli, Linping Hui, Qingfu Zhang, Xiupeng Zhang, Enhua Wang, Limei Sun, Xueshan Qiu

**Affiliations:** ^1^ Department of Pathology, College of Basic Medical Sciences and First Affiliated Hospital, China Medical University, Shenyang, China; ^2^ Fouth Affiliated Hospital, China Medical University, Shenyang, China

**Keywords:** tripartite motif-containing 71, non-small cell lung cancer, inhibitor of kappaB, nuclear factor kappaB, ubiquitination

## Abstract

Tripartite motif-containing (TRIM) 71 belongs to the TRIM protein family. Many studies have shown that TRIM71 plays conserved roles in stem cell proliferation, differentiation, and embryonic development; however, the relationship between TRIM71 and tumorigenesis is not clear. In this study, we demonstrate that TRIM71 expression in non-small cell lung cancer (NSCLC) is associated with tumor size, lymph node metastasis, TNM stage, and poor prognosis. We found that TRIM71 was highly expressed in NSCLC cell lines compared with that in human normal bronchial epithelial cells. Moreover, by altering the expression of TRIM71 in selected cell lines, we found that TRIM71 promoted the proliferation of NSCLC cells through activation of the inhibitor of kappaB/nuclear factor kappaB pathway. These results suggested that TRIM71 plays a role in promoting the development of NSCLC.

## INTRODUCTION

Lung cancer is one of the leading causes of all cancer-related deaths worldwide, and the incidence is only increasing. Most cases of lung cancer are non-small cell lung cancer (NSCLC). Although many treatment approaches have been established, long-term survival rates in patients with lung cancer is still not optimal [1]. With the rise of immunotherapy and molecular targeted therapy, further improvement in our understanding of the molecular processes of pulmonary carcinogenesis, potential biomarkers, and novel therapeutic targets is needed.

Tripartite motif-containing 71 (TRIM; also known as lineage variant 41) belongs to the TRIM protein family and is a highly conserved target of *let-7* [2–7]. TRIM family members all contain a similar RBCC structural sequence. RBCC proteins participate extensively in embryonic development, tumorigenesis, and inflammatory reactions [8]. The RBCC structure contains a RING domain, one or two b-box domains, and a coiled-coil domain [9–13]. In addition to the RBCC sequence, TRIM71 contains six repeats of the NHL domain.

The RING motif of TRIM71 is essential for target protein ubiquitin-dependent degradation or stabilization. TRIM71 acts as a specific E3 ubiquitin ligase, downregulates the expression of AGO proteins [14, 15] and the RNA-induced silencing complex components, and promotes the expression of *let-7* downstream target proteins [16–18]. Moreover, TRIM71 has been shown to stabilize Shc SH2-binding protein 1 (SHCBP1), a component of fibroblast growth factor (FGF) signaling, via ubiquitination and to enhance FGF signaling in neuronal progenitor cells [19].

Studies have shown that TRIM71 plays an important role in biological development. TRIM71 regulates the growth of embryos, promotes proliferation, and inhibits maturation and differentiation in embryonic stem cells during embryonic development in *Caenorhabditis elegans* [20–24], mice [25, 26], and chickens [27–33] and stimulates the proliferation of hepatocellular carcinoma cells [15]. Moreover, a previous report demonstrated that exogenous TRIM71 expression promotes the formation of inducible pluripotent stem cells (iPSCs), whereas knockdown of endogenous TRIM71 expression inhibits reprogramming [34]. Although TRIM71 has been shown to promote proliferation in a variety of cells, the relationship between TRIM71 and tumor biology and the associated molecular mechanisms are still unclear.

Accordingly, in this study, we examined the expression of TRIM71 in NSCLC tissues and cell lines by immunohistochemistry and western blotting. Moreover, we altered the expression of TRIM71 in NSCLC cell lines and evaluated changes in cancer-related phenotypes in these cells to determine the role of TRIM71 in NSCLC.

## RESULTS

### Patient demographics and clinicopathological characteristics

A total of 282 patients were enrolled in this study. Patients (178 men and 104 women) were between 30 and 79 years of age. According to the seventh edition of the International Union against Cancer (UICC) TNM Staging System for Lung Cancer, patients were categorized as having stage I (*n =* 97), II (*n =* 93), and III (*n =* 92) disease. The histological diagnoses and differentiation grades of the tissue samples were evaluated according to the WHO classification system as squamous cell carcinoma (*n =* 141) and adenocarcinoma (*n =* 141). Additionally, 107 samples were highly differentiated, and 175 cases were moderately or poorly differentiated. Lymph node metastases were present in 129 cases and absent in 153 cases.

### TRIM71 is highly expressed in NSCLC and correlated with poor prognosis

**Figure 1:**
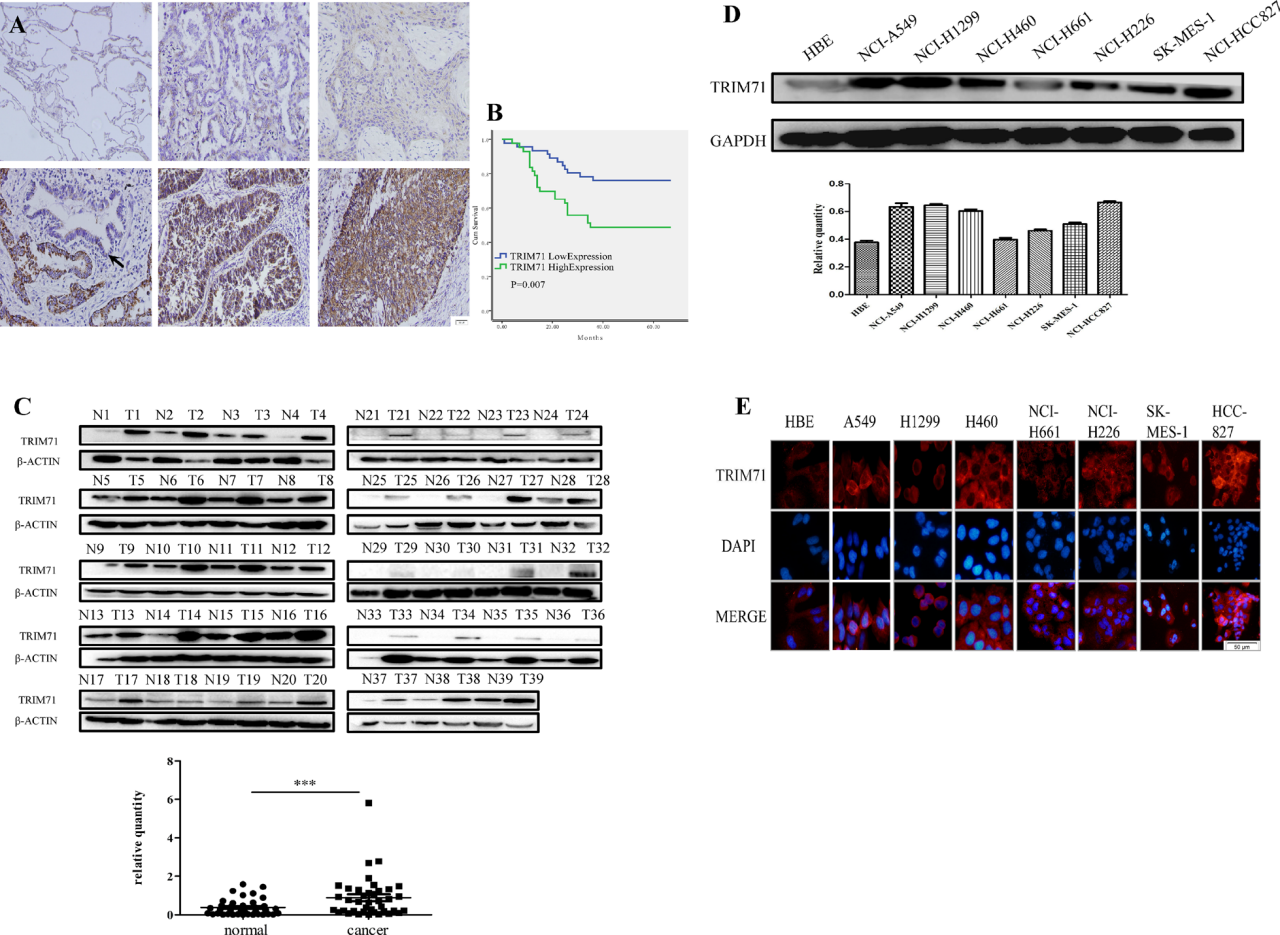
TRIM71 is highly expressed in non-small cell lung cancer and is associated with poor clinical prognosis (**A**) Expression of TRIM71 in normal alveolar tissues is negative, low and high expression of TRIM71 in lung squamous cell carcinoma and adenocarcinoma are shown in the following panels. Normal lung and bronchus epithelium (arrow) showed that the expression of TRIM71 was negative. (**B**) Kaplan-Meier survival analysis in 89 patients according to TRIM71 expression. Patients with high TRIM71 expression had significantly lower survival rates than those with low TRIM71 expression. *p* < 0.007. (**C**) Western blotting was performed to detect TRIM71 protein in paired fresh lung cancerous tissues (T) and adjacent nontumorous lung tissues (N), marked with serial numbers. Relative quantification of protein expression via grayscale values showed differences of TRIM71 expression between paired fresh lung cancerous tissues and adjacent nontumorous lung tissues was statistically significant. ^***^p < 0.001. (**D**) Expression levels of TRIM71 in HBE cells and seven NSCLC cell lines, as determined by western blot analysis. Relative quantification analysis base on grayscale values. (**E**) Immunofluorescence assays were performed to detect the expression of TRIM71 in human normal bronchial epithelial cell lines and non-small cell lung cancer cell lines; TRIM71 was located in the cytoplasm.

Previous studies have reported that TRIM71 promotes stem cell proliferation and may contribute to the induction of iPSCs. We hypothesized that TRIM71 also plays a role in the proliferation of NSCLC cells. Therefore, we performed immunohistochemistry of 282 NSCLC tissues and 30 normal lung tissues. In lung squamous cell carcinoma and adenocarcinoma, TRIM71 was localized in the cytoplasm and was significantly upregulated compared with that in normal lung tissues, alveoli, and bronchi tissues. In normal lung tissues, TRIM71 expression was very low or absent; in contrast, of the 282 cases of lung squamous cell carcinoma and adenocarcinoma, only 39 were TRIM71-negative (Figure 1A).

Importantly, TRIM71 expression was found to be positively correlated with clinicopathological parameters, including differentiation (*p* = 0.016), tumor size (*p* < 0.0001), lymph node metastasis (*p* = 0.007), and pathological TNM stage (*p* = 0.01). The correlations between TRIM71 expression and age (*p* = 0.311), sex (*p* = 0.142), and histological type (*p* = 0.06) were not statistically significant (Table 1). Moreover, Kaplan-Meier survival analysis of TRIM71-negative and -positive cases in 89 patients with complete follow-up data showed that high levels of TRIM71 were significantly associated with poor patient outcomes (*p* < 0.05; Figure 1B).

**Table 1:** Correlationship between TRIM71 expresstion and clinicopathologal factors in 282 cases of NSCLC

		number of patients	TRIM71 negtive	TRIM71 positive	*P* value
age					
	≥ 60	140	60	80	0.311
	< 60	142	66	76	
gender					
	male	178	81	97	0.142
	female	104	55	49	
histology					
	squamous carcinoma	141	56	85	0.06
	adenocarcinoma	141	70	71	
differentiation					
	Poor-moderate	175	69	106	0.016
	well	107	57	50	
size					
	≥ 3	163	38	125	0.000
	< 3	119	88	31	
lymph node status					
	N0	153	79	74	0.007
	N1 + N2 + N3	129	47	82	
pTNM stage					
	I + II	190	101	89	0.01
	III	92	25	67	

Additionally, we collected 39 pairs of fresh NSCLC and paracancerous tissues and evaluated TRIM71 protein levels by western blotting. In tumor tissues, TRIM71 protein levels were generally higher than that in adjacent lung tissues (Figure 1C). Notably, the differences in TRIM71 protein expression between tumor tissues and adjacent lung tissues were statistically significant (*p* < 0.0001).

We then performed western blotting to detect TRIM71 protein expression in human normal bronchial epithelial cells and commonly used NSCLC cell lines. We found that TRIM71 protein levels were higher in tumor cell lines than in HBE normal bronchial epithelial cells (Figure 1D). These findings were further supported by immunofluorescence results (Figure 1E).

These findings suggests that TRIM71 expression is generally high in NSCLC tissues and TRIM71 is likely to play a role in the development of NSCLC.

### TRIM71 protein expression promotes the proliferation of NSCLC cells

On the basis of the above findings, we next aimed to further determine the role of high TRIM71 protein expression in NSCLC. To this end, we altered TRIM71 expression by downregulation or upregulation in NCI-A549 and NCI-H1299 cell lines, using shRNA or the pcmv-flag TRIM71 vector, respectively.

**Figure 2:**
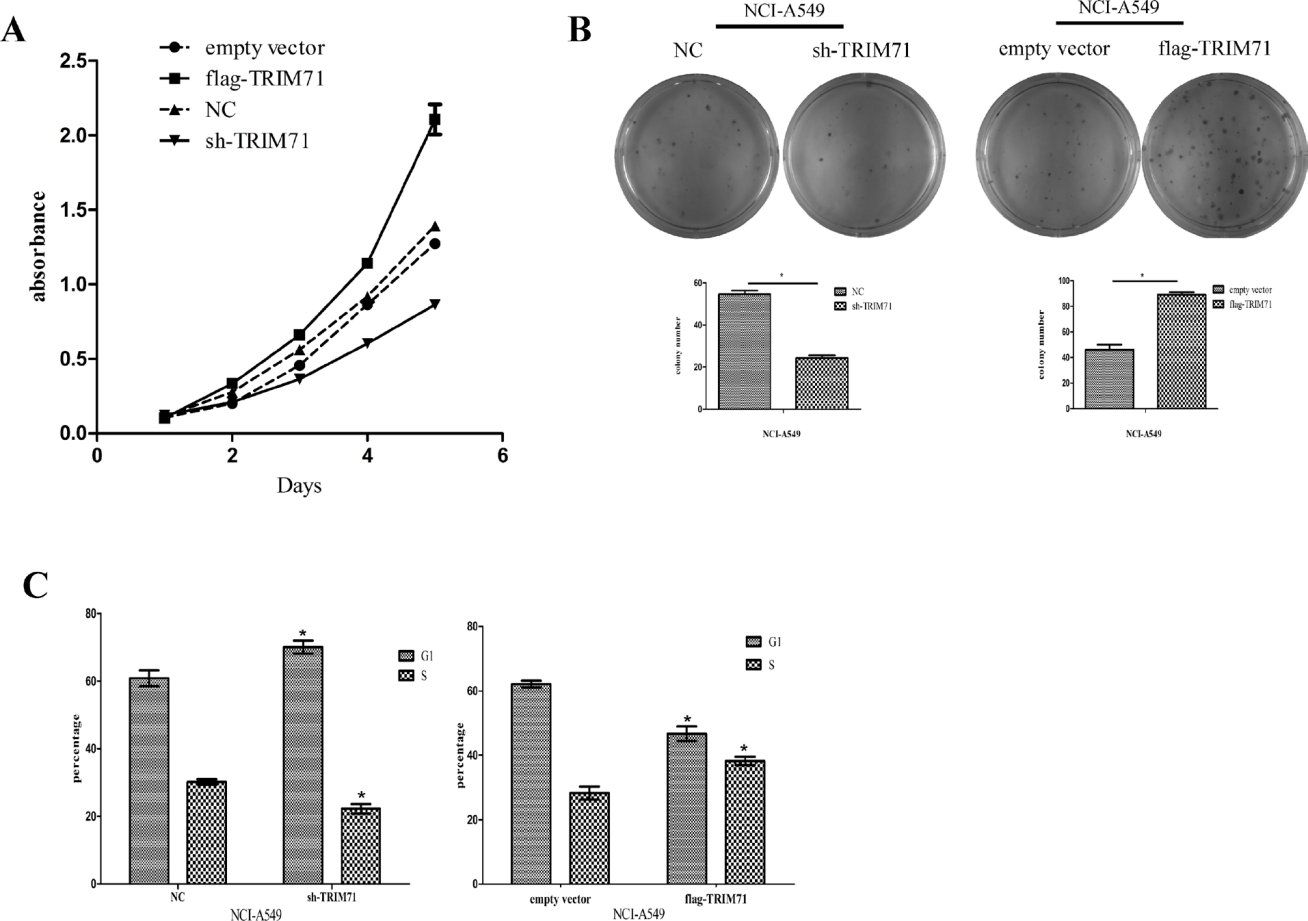
TRIM71 protein expression is positively correlated with the proliferation of non-small cell lung cancer cells (**A**) MTT assays were performed to assess cell proliferation in the context of TRIM71 overexpression and downregulation. Overexpression of TRIM71 in NCI-A549 cells enhanced cell proliferation, and knockdown of TRIM71 caused growth inhibitory effects. (**B**) Colony formation was assessed in cells with TRIM71 overexpression or downregulation. Overexpression of TRIM71 significantly enhanced colony formation and vice versa. ^*^p < 0.05. (**C**) Flow cytometry was performed to assess the effects of TRIM71 overexpression or downregulation on cell cycle distribution. ^*^p < 0.05.

MTT cell proliferation assays showed that high expression of TRIM71 enhanced cell proliferation activity, whereas downregulation of TRIM71 inhibited cell viability (Figure 2A). Subsequent colony-formation assays showed positive correlations between TRIM71 expression and the colony-formation ability of tumor cells (Figure 2B). The cell cycle distribution was then analyzed by flow cytometry. We found that high expression of TRIM71 promoted the G_1_/S transition, whereas decreased TRIM71 levels inhibited the G_1_/S phase transition (Figure 2C).

Similar results were obtained in NCI-H1299 (Supplementary Figure 1) and NCI-A549 cells. Thus, these data suggested that TRIM71 overexpression promotes the proliferation of NSCLC cells, consistent with our immunohistochemical results in 282 cases of NSCLC showing that TRIM71 is associated with tumor size.

### TRIM71 regulates the proliferation of NSCLC cells through the IκB-α/NF-κB pathway

**Figure 3:**
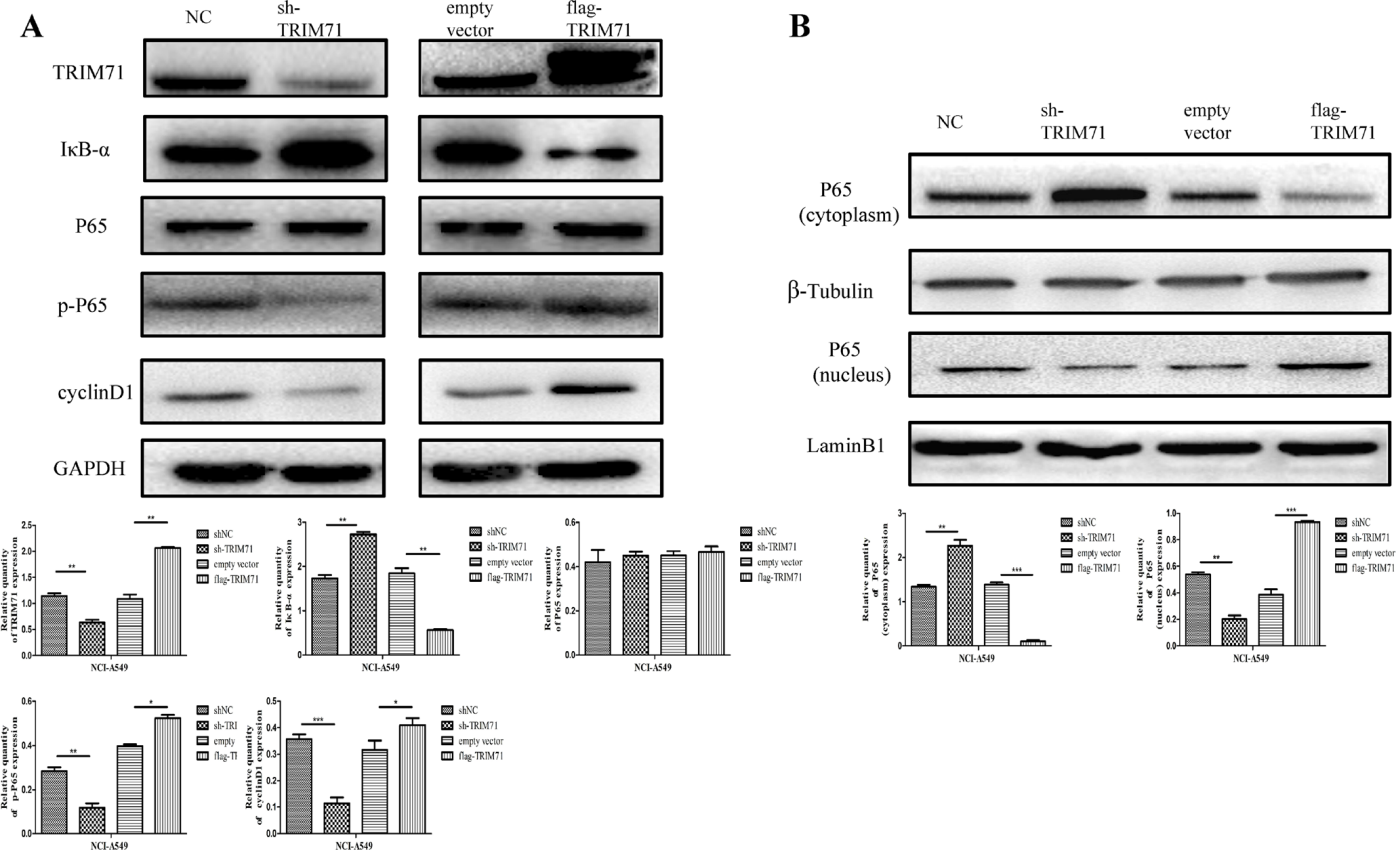
TRIM71 affects the proliferation of non-small cell lung cancer through the IκB-α/NF-κB pathway (**A**) Effects of TRIM71 expression on the expression of cyclin D1,P65, and IκB-α. Overexpression of TRIM71 resulted in a reduction of IκB-α protein level, and upregulation of cyclin D1, p-P65 protein levels. Knockdown of TRIM71 enhanced the expression IκB-α protein level, and reduced cyclin D1, p-P65 protein levels. Quantification of western blotting data using three independent blots. ^*^p < 0.05, ^**^p < 0.01. (**B**) Effects of TRIM71 expression on the phosphorylation and expression of p65 in nuclear extracts. Overexpression of TRIM71 reduced IκB-α protein level and increased p65 phosphorylation and nuclear translocation and vice versa. ^*^p < 0.05, ^**^p < 0.01, ^***^p < 0.001.

On the basis of the observed relationship between tumor size and TRIM71 expression in clinical cases and the functions of TRIM71 in NSCLC cell proliferation, we explored the specific molecular biological mechanisms associated with these effects. After upregulation or downregulation of TRIM71 protein levels, a series of proliferation-related proteins were detected and verified via western blotting. The results showed that the expression of cyclin D1 was reduced after TRIM71 downregulation, whereas overexpression of TRIM71 increased the level of cyclin D1. Moreover, IκB-α protein levels were negatively correlated with TRIM71 protein levels (Figure 3A) in a concentration-dependent manner (Supplementary Figure 2B).

**Figure 4:**
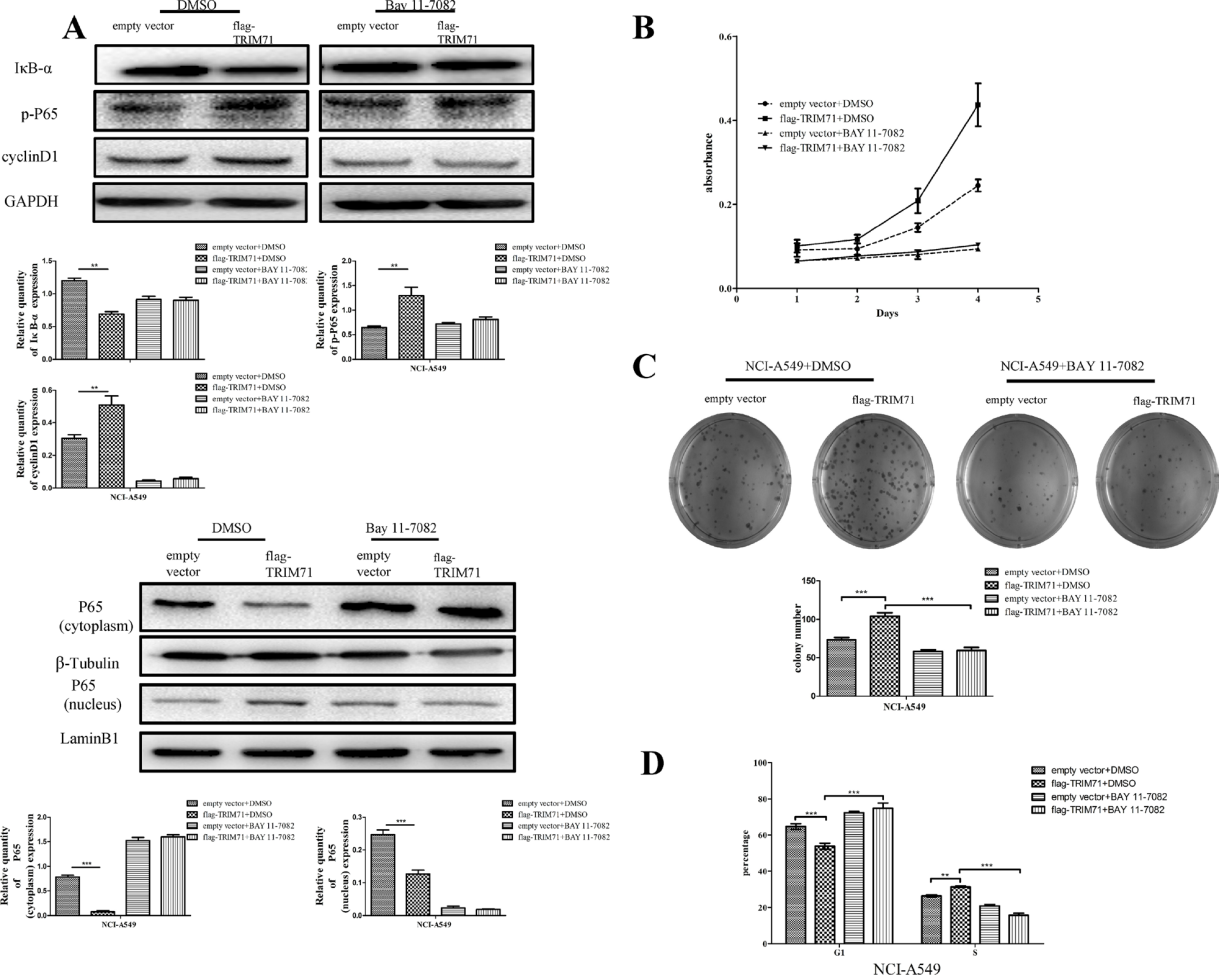
TRIM71 regulates the proliferation of non-small cell lung cancer cells through the IκB-α/NF-κB pathway (**A**) Cells were treated with the NF-κB pathway inhibitor BAY 11-7082 or DMSO as a control, and the effects of TRIM71 expression on the expression of cyclin D1 and the phosphorylation of p65 were evaluated. Correlation between TRIM71 and these proteins was canceled after using the NF-κB inhibitor BAY 11-7082. ^**^p < 0.005, ^***^p < 0.001. (**B**) MTT assays were performed to evaluate the effects of TRIM71 expression and the NF-κB inhibitor BAY 11-7082 on cell proliferation. (**C**) Colony formation assays were performed to evaluate the effects of TRIM71 expression and the NF-κB inhibitor BAY 11-7082 on colony formation. ^***^p < 0.001. (**D**) Flow cytometry was performed to evaluate the effects of TRIM71 expression and the NF-κB inhibitor BAY 11-7082 on the G_1_/S phase transition. ^*^p < 0.01, ^***^p < 0.001.

Next, we evaluated the effects of TRIM71 expression on activation of the NF-κB pathway, using nuclear extracts of NSCLC cells. The results showed that overexpression of TRIM71 reduced IκB-α protein levels and increased p65 phosphorylation (Figure 3A) and nuclear translocation (Figure 3B). Thus, increased expression of TRIM71 promotes NF-κB pathway activity. Next, we evaluated the effects of the NF-κB pathway by using inhibitor BAY 11-7082, which inhibits IκB-α phosphorylation and ubiquitin-dependent degradation, resulting in p65 release. The negative correlation between TRIM71 and IκB-α was canceled using the NF-κB inhibitor BAY 11-7082 (Figure 4A). Moreover, MTT assays, colony formation assays, and flow cytometry showed that BAY 11-7082 significantly inhibited the ability of TRIM71 to enhance colony formation, proliferation, and the G_1_/S phase transition in NSCLC cells (Figure 4B–4D). These results suggested that activation of the IκB-α/NF-κB pathway is involved in the contribution of TRIM71 to the proliferation of NSCLC. Similar results were also obtained in NCI-H1299 cells (Supplementary Figure 3).

### TRIM71 is involved in IκB-α ubiquitin-dependent degradation through its RING domain

Finally, we evaluated how TRIM71 interacts with IκB-α and activates the NF-κB pathway to promote cell proliferation. In the cytoplasm, IκB-α combines with NF-κB and blocks the NF-κB protein nuclear localization sequence, thereby inhibiting NF-κB transcription factor activity. Subsequently, IκB-α is degraded via the ubiquitination pathway after phosphorylation and release of NF-κB/p65. Phosphorylated p65 is then translocated to the nucleus, where it functions as a transcription factor. However, BAY 11-7082 blocks IκB-α phosphorylation and thereby inhibits the NF-κB pathway.

**Figure 5:**
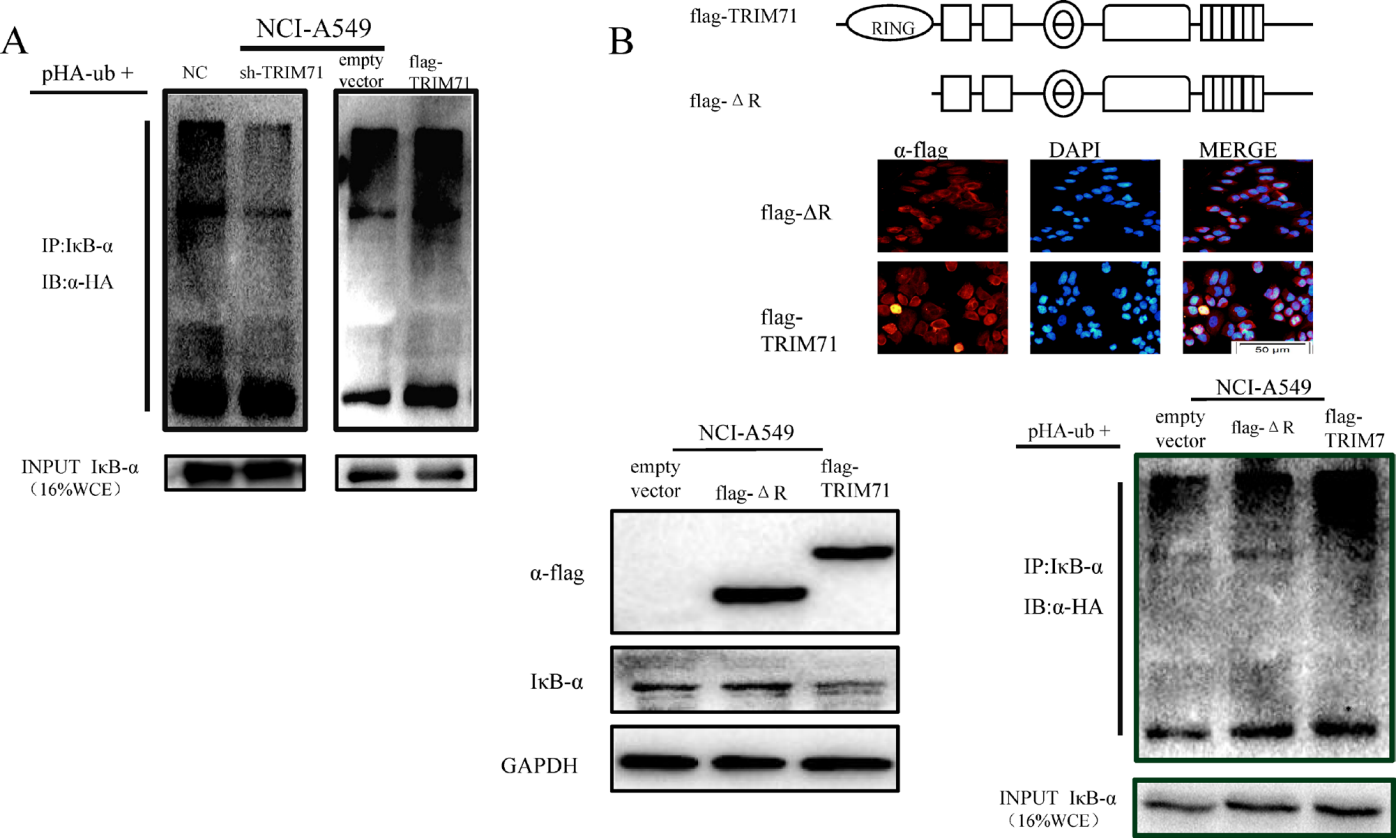
TRIM71 is involved in IκB-α ubiquitination and degradation through its ring finger structure, affecting the NF-κB pathway (**A**) Effects of TRIM71 expression on the ubiquitination of IκB-α. Flag-tagged wild-type of TRIM71 was expressed in cells along with HA-ubiquitin (Ub). The levels of IκB-α ubiquitylation were evaluated by the immunoprecipitation of IκB-α using anti-IκB-α antibodies followed by anti-HA immunoblotting. (**B**) Immunofluorescence staining showed the expression and localization of the transfected pcmv-flag TRIM71 plasmid with deletion of the RING domain and pcmv-flag TRIM71 plasmid. Cells were transfected with the pcmv-flag TRIM71 plasmid and pcmv-flag TRIM71 with deletion of the RING domain, and immunoblotting was performed determine the effects of RING-deleted TRIM71 on IκB-α protein levels. Ubiquitination assays of IκB-α. Flag-tagged wild-type or RING finger domain-deleted mutants (ΔR) of TRIM71 were expressed in cells along with HA-ubiquitin (Ub). The levels of IκB-α ubiquitylation were evaluated by immunoprecipitation of IκB-α using anti-IκB-α antibodies followed by anti-HA immunoblotting. TRIM71 did not reduce the expression of IκB-α or increase the ubiquitination level of IκB-α after deletion of the RING domain.

Reports have shown that the RING domain of TRIM71 has typical E3 ubiquitin ligase activity. Thus, we hypothesized that TRIM71 may contribute to activation of the NF-κB pathway by increasing the level of ubiquitination of IκB-α. Accordingly, we transfected flag-tagged wild-type TRIM71 into NCI-A549 cells along with HA-ubiquitin (Ub) and then treated cells with the 26S proteasome inhibitor MG132. The levels of IκB-α ubiquitylation were evaluated by the immunoprecipitation of IκB-α, using anti-IκB-α antibodies followed by anti-HA immunoblotting. The results revealed a positive correlation between TRIM71 levels and IκB-α ubiquitination (Figure 5A). We also transfected cells with the RING domain-deleted pcmv-flag TRIM71 ΔR plasmid and found that when the RING domain was deleted, the effects of TRIM71 on IκB-α were reversed, and IκB-α protein levels were not decreased. Flag-tagged wild-type or RING finger domain-deleted mutants (ΔR) of TRIM71 were expressed in NCI-A549 cells along with HA-ubiquitin (Ub). The levels of IκB-α ubiquitylation were evaluated by the immunoprecipitation of IκB-α, using anti-IκB-α antibodies followed by anti-HA immunoblotting. TRIM71 did not reduce the expression of IκB-α or increase the ubiquitination level of IκB-α after deletion of the RING domain (Figure 5B). These data suggested that TRIM71 is involved in the ubiquitin-dependent degradation of IκB-α through its RING domain to activate the NF-κB pathway. Similar results were also obtained in NCI-H1299 cells (Supplementary Figure 4).

## DISCUSSION

In this study, we found that TRIM71 protein levels are correlated with a range of clinicopathological parameters such as tumor size, differentiation, lymph node metastasis, TNM staging, and prognosis. Notably, we also showed that TRIM71 expression differs between cancer tissues and adjacent tissues, with overexpression observed in NSCLC cells. Similar trends were observed in the human normal bronchial cell line HBE and seven NSCLC cell lines. We also found that TRIM71 promoted cell cycle progression, proliferation, and colony formation in NSCLC cells. On the basis of these findings, we then examined a number of proliferation-related genes known to be associated with TRIM71.

Although TRIM71 has been shown to degrade AGO protein [14, 15], in our study we found no such interaction, similar to the results of a previous study [19]. Further studies are needed to evaluate the mechanisms mediating these effects.

Moreover, we found that TRIM71 altered the ubiquitination of other proteins such as IκB-α in NSCLC and promoted the ubiquitin-dependent degradation of IκB-α, resulting in activation of the NF-κB pathway and stimulation of downstream gene transcription. Although our study did not adequately demonstrate whether TRIM71 interacted directly with IκB-α to promote its ubiquitination, and the role of phosphorylated AKT in IκB-α protein expression is still unclear, we did verify that the IκB-α/NF-κB pathway plays an important role in TRIM71-dependent proliferation of NSCLC cells. Thus, in contrast to previous studies, our findings suggested that TRIM71 may participate in different biological mechanisms in cancer cells than in embryos and embryonic stem cells. Although further studies are needed to determine how TRIM71 functions in these biological processes, our results provide important insights into the roles and mechanisms of TRIM71 in tumorigenesis.

## MATERIALS AND METHODS

### Tissue samples and patient data

Tumor specimens from 282 patients with NSCLC who underwent complete surgical resection at the First Affiliated Hospital of China Medical University between 2010 and 2015 were selected from the Department of Pathology and retrospectively analyzed. Of the 282 patients with lung cancer, complete follow-up data were available in 89 cases. Patient survival was defined as the time from the date of surgery to the end of the follow-up period or to the date of death due to recurrence or metastasis. None of the patients underwent radiotherapy or chemotherapy before surgical resection. We also collected 30 adjacent tissue specimens as negative controls from the same patients. For protein analysis, 39 freshly isolated specimens, including both tumor tissues and corresponding normal adjacent tissues, were stored at −80°C after resection for subsequent protein extraction.

### Immunohistochemistry

All specimens were fixed in 10% neutral formalin, embedded in paraffin, and prepared as 4-μm-thick serial sections. Immunostaining was carried out according to the streptavidin-peroxidase method. The sections were incubated with anti-TRIM71 rabbit polyclonal antibodies (1:1000 dilution; PAB19293; Abnova, Taiwan, China) at 4°C overnight. Sections were washed in phosphate-buffered saline (PBS) and incubated with reagents A and B (EliVsion Reagent; KIT9921; MaiXin, Fuzhou, China), according to the manufacturer’s instructions. Sections were then developed using 3,3-diaminobenzidine tetrahydrochloride (MaiXin), lightly counter-stained with hematoxylin, dehydrated in alcohol, and mounted. Two investigators blinded to the clinical data semiquantitatively scored all slides by evaluating the staining intensity and percentages of cells stained in representative areas of each slide. The percentages of stained cells were scored as follows: 1 (1–25%), 2 (26–50%), 3 (51–75%), or 4 (76–100%). Based on the intensity of staining, the expression of TRIM71 was also scored as follows: 0 (no staining), 1 (light yellow granules), and 2 (deep yellow or brown particles). In each tumor sample, the scores of the staining intensity were multiplied by the percentage of positive cells stained to give a final score between 0 and 8. Since the scores for all 38 normal bronchial epithelial were less than 3, a score of 3 was set as the baseline; cases with a score of less than 3 were considered as negative expression (normal expression), and cases with score greater than 3 were defined as positive expression (high expression).

### Cell culture

All cell lines were purchased from Shanghai Cell Bank (Shanghai, China) and cultured in medium containing 10% qualified fetal calf serum (FB15015; Clark Biosciences, Richmond, VA, USA), 100 IU/mL penicillin (Sigma, St. Louis, MO, USA), and 100 μg/mL streptomycin (Sigma). HBE cells were cultured in Dulbecco’s modified Eagle’s medium (DMEM) with high glucose; NCI-A549 cells were cultured in F12K medium; NCI-H1299, NCI-H460, NCI-H226, NCI-H661, and NCI-HCC827 cells were cultured in RPMI 1640 medium; and SK-MES-1 cells were cultured in MEM medium. Cells were grown in sterile culture flasks and plates (Thermo Fisher Scientific, USA).

### Plasmid construction and transfection

pcmv-flag-Trim71 and pcmv-flag-Trim71 ΔR plasmids were a gift from Dr. Gregory (Children’s Hospital Boston, Department of Biological Chemistry and Molecular Pharmacology, Harvard Medical School, Harvard Stem Cell Institute, Boston, MA, USA). Short hairpin RNA (shRNA) targeting TRIM71 was cloned into the GV248 vector by GENECHEM Co. Ltd. (Shanghai, China). The sequences were as follows:

TRIM71-RNAi (33820-1)-a, 5′-CcggggTCACTGACTTCAACAACCACTCGAGTGGTTGTTGAAGTCAGTGACCTTTTTg-3′; TRIM71-RNAi (33820-1)-b, 5′-aattcaaaaaggTCACTGACTTCAACAACCACTCGAGTGGTTGTTGAAGTCAGTGACC-3′. The plasmids were transfected into the cells using Lipofectamine 3000 (Life-L3000015; Invitrogen, Carlsbad, CA, USA) according to the manufacturers’ protocol.

### Western blotting

Total cellular protein was extracted using lysis buffer (P0013; Beyotime Biosciences, Shanghai, China) with protease inhibitor cocktail (B14002; Biotool, Shanghai, China) with or without phosphatase inhibitor cocktail (B15002; Biotool) according to the manufacturer’s instructions and quantified by the Bradford method. Sixty micrograms of protein was separated by sodium dodecyl sulfate polyacrylamide gel electrophoresis on 10% gels. After transfer to polyvinylidene fluoride membranes (Millipore, Billerica, MA, USA), the membranes were blocked in 5% skim milk (232100; BD-Difco, USA) in TBST at room temperature, incubated overnight at 4°C with anti-TRIM71 (1:1000 dilution; PAB19293, Abnova, Taiwan, China), anti-p65 (SC-372; Santa Cruz Biotechnology),anti-IκB-α (SC-371; 1:100 dilution; Santa Cruz Biotechnology), anti-cyclin D1 (60186-1-Ig), anti-phosphorylated-p65 (3033T; Cell Signaling Technology), anti-β-tubulin (HT101; Transgen BioTech, Beijing, China), anti-HA (HT301; Transgen BioTech), anti-flag (HT201; 1:500 dilution; Transgen BioTech), anti-laminin B1 (sc-6216; Santa Cruz Biotechnology), anti-β-actin (sc-1616; Santa Cruz Biotechnology), and anti-glyceraldehyde-3-phosphate dehydrogenase (GAPDH; 1:2000 dilution, TA-08; ZSGB-BIO, Beijing, China) antibodies. Membranes were washed and incubated with horseradish peroxidase (HRP)-conjugated anti-mouse/rabbit IgG (1:2000 dilution, ZSGB-BIO) at room temperature for 2 h, and the protein bands were visualized using SuperSignal West Pico Chemiluminescent Substrate (ECL 34080; Thermo Scientific, Waltham, MA, USA) and quantified using a BioImaging System (UVP, Upland, CA, USA). The relative protein levels were calculated using β-actin or GAPDH protein as an endogenous control.

### 3-(4,5-Dimethylthiazol-2-yl)-2,5-diphenyl tetrazolium bromide (MTT) cell proliferation assays

Cells were plated in 96-well plates in medium containing 10% FBS at about 3000 cells/well at 24 h after transfection. For quantification of cell viability, cultures were stained after 4 days by MTT assays. Briefly, 50 μL of 5 mg/mL MTT solution was added to each well, and samples were incubated for 4 h at 37°C. The medium was then removed from each well, and the resulting MTT formazan product was solubilized in 150 μL dimethylsulfoxide (DMSO). The results were quantified spectrophotometrically by evaluation of the absorbance after MTT treatment at a wavelength of 490 nm using a microplate reader (Model 550; Bio-Rad, Hercules, CA, USA).

### Colony formation assays

Cells were transfected with plasmids or shRNA for 24 h, and 300–500 cells per group were plated in 6-well plates and incubated for 10 days or so until visible colonies formed. The colonies were fixed with methanol, stained with hematoxylin, and scored according to the number of the colonies after the plates air dried.

### Flow cytometry

Forty-eight hours after transfection, we collected the cells from 6-well plates by centrifuging, washed the cells in PBS, and incubated cells with 70% ethanol overnight at −20 r. Cells were then collected by centrifugation, washed in PBS, and incubated with 300–500 μL propidium iodide (PI; Beyotime Biosciences) and RNase for 30 min. Cells were then subjected to flow cytometry analysis using Mod Fit 1.0.

### Cellular fractionation

Nuclear and cytoplasmic fractions were isolated using a Nuclear and Cytoplasmic Extraction kit (P0028; Beyotime Biosciences) according to the manufacturer’s instructions.

### Proteasome inhibition assays, ubiquitination assays, and immunoprecipitation

For proteasome inhibition assays, cells were transfected with the indicated plasmids (Figures 4 and 5B). At 48 h after transfection, the 26S proteasome inhibitor MG132 (s1748; Beyotime Biotechnology) was added at a final concentration of 10 μM for 5 h, after which samples were collected. Cells were lysed in lysis buffer, and cell debris was pelleted by centrifugation at 12,000 rpm for 10 min at 4°C. Supernatants were collected for IP. IP was carried out using whole cell lysates (approximately 200 μg protein) with 4–10 μg antibody and 20 μL protein A/G agarose (P2012; Beyotime Biosciences). The cell lysates were precleared with 20 μL agarose A/G beads by rocking for 1 h at 4°C. Beads were removed, and appropriate antibodies were added. Samples were then incubated with 20 μL agarose A/G beads by rocking for 4–6 h at 4°C, and lysates were then added, followed by incubation with rocking overnight at 4°C. The immune complexes were collected by centrifugation, followed by washing in cell lysis buffer before analysis. The levels of IκB-α ubiquitylation were evaluated by immunoprecipitation of IκB-α using anti-IκB-α antibodies followed by anti-HA immunoblotting.

### Inhibitors

For mechanistic analysis, we used the NF-κB pathway inhibitor BAY 11-7082 (s2913; Selleck Chemicals, Shanghai, China) at a final concentration of 5 μM for 6 h. Additionally, we used the 26S proteasome inhibitor MG132 (s1748; Beyotime Biotechnology) at a final concentration of 10 μM for 5 h.

### Immunofluorescence staining

The cells were cultured on glass coverslips in 24-well plates, fixed with 4% paraformaldehyde for 15 min, and then permeabilized with 0.1% Triton X-100 for 10 min. After washing with PBS, cells were blocked with nonimmune goat serum for 30 min at 25°C and then incubated with LIN41 antibody (SC-134793; 1:100 dilution; Santa Cruz Biotechnology) or anti-Flag antibodies (HT201; 1:100 dilution; Transgen BioTech) overnight at 4°C. Cells were then incubated with rhodamine (TRITC)-conjugated goat anti-mouse antibodies (ZF-0313; 1:100 dilution; ZSGB-BIO), counterstained with DAPI, and mounted.

### Statistical analysis

SPSS 17.0 for Windows was used for all statistical analyses. The Kaplan-Meier method was applied for survival curves, and log-rank tests were performed for comparisons. Student’s *t*-tests were performed to analyze the statistical significance of differences between TRIM71 expression and clinical parameters. Differences with *p* values of less than 0.05 were considered statistically significant.

### Ethics

This study was conducted with the approval of the Ethics Review Committee of the First Affiliated Hospital of China Medical University. Fresh samples and paraffin-embedded NSCLC samples were obtained from patients admitted to the Pathological Department of the First Affiliated Hospital of China Medical University. Informed consent was obtained from all patients. Two pathologists performed the pathologic diagnoses independently based on World Health Organization (WHO) guidelines.

## SUPPLEMENTARY MATERIALS FIGURES


