# Supplementary material for: E3 ubiquitin ligase tripartite motif-containing 71 promotes the proliferation of non-small cell lung cancer through the inhibitor of kappaB-α/nuclear factor kappaB pathway
Source: Oncotarget. 2017 Jul 7;9(13):10880–90. doi: 10.18632/oncotarget.19075 (PMC5834285; doi:10.18632/oncotarget.19075)
Supplement: Supplementary file 1 [file oncotarget-09-10880-s001.pdf]

E3 ubiquitin ligase tripartite motif-containing 71 promotes the proliferation of non-small cell lung cancer through the inhibitor of kappaB-α/nuclear factor kappaB pathway

SUPPLEMENTARY MATERIALS

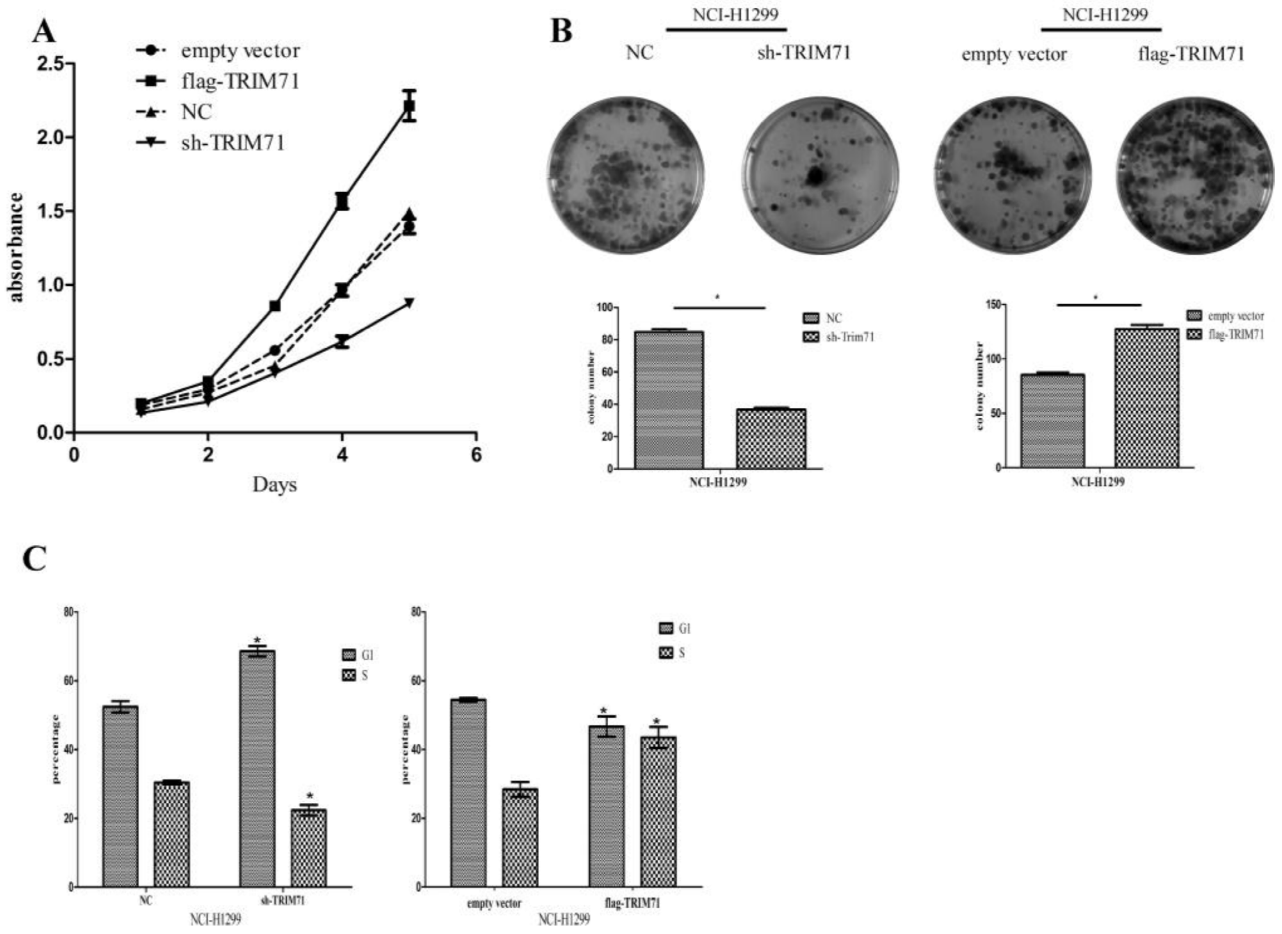

Supplementary Figure 1: TRIM71 protein expression is positively correlated with the proliferation of non-small cell lung cancer cells. (**A**) MTT assays were performed to assess cell proliferation in the context of TRIM71 overexpression and downregulation. Overexpression of TRIM71 in NCI-H1299 cells enhanced cell proliferation, and knockdown of TRIM71 caused growth inhibitory effects. $^{*}p < 0.05$. (**B**) Colony formation was assessed in cells with TRIM71 overexpression or downregulation. Overexpression of TRIM71 significantly enhanced colony formation and vice versa. $^{*}p < 0.05$. (**C**) Flow cytometry was performed to assess the effects of TRIM71 overexpression or downregulation on cell cycle distribution. $^{*}p < 0.05$.

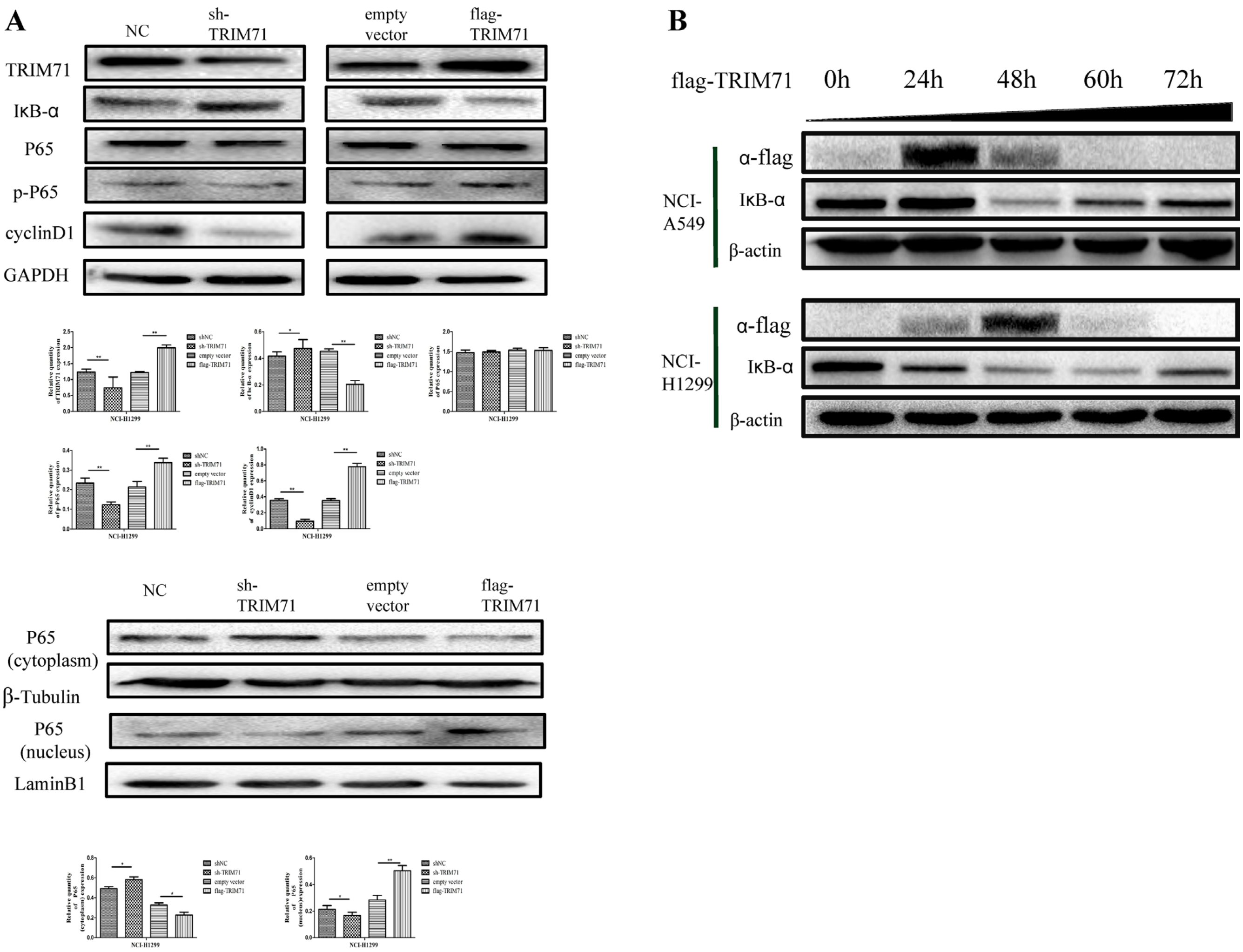

Supplementary Figure 2: TRIM71 affects the proliferation of non-small cell lung cancer through the IκB-α/NF-κB pathway. (**A**) Effects of TRIM71 on the expression of cyclin D1, P65, and IκB-α. Overexpression of TRIM71 resulted in a reduction of IκB-α protein level, and upregulation of cyclin D1, p-P65 protein levels. Knockdown of TRIM71 enhanced the expression IκB-α protein level, and reduced cyclin D1, p-P65 protein levels in NCI-H1299 cells. Quantification of western blotting data using three independent blots. Effects of TRIM71 expression on the phosphorylation and expression of p65 in nuclear extracts.Overexpression of TRIM71 reduced IκB-α protein level and increased p65 phosphorylation and nuclear translocation and vice versa in NCI-H1299 cells. $^{*}p < 0.05$, $^{**}p < 0.01$. (**B**) IκB-α protein levels were negatively correlated with TRIM71 protein levels in a concentration-dependent manner.

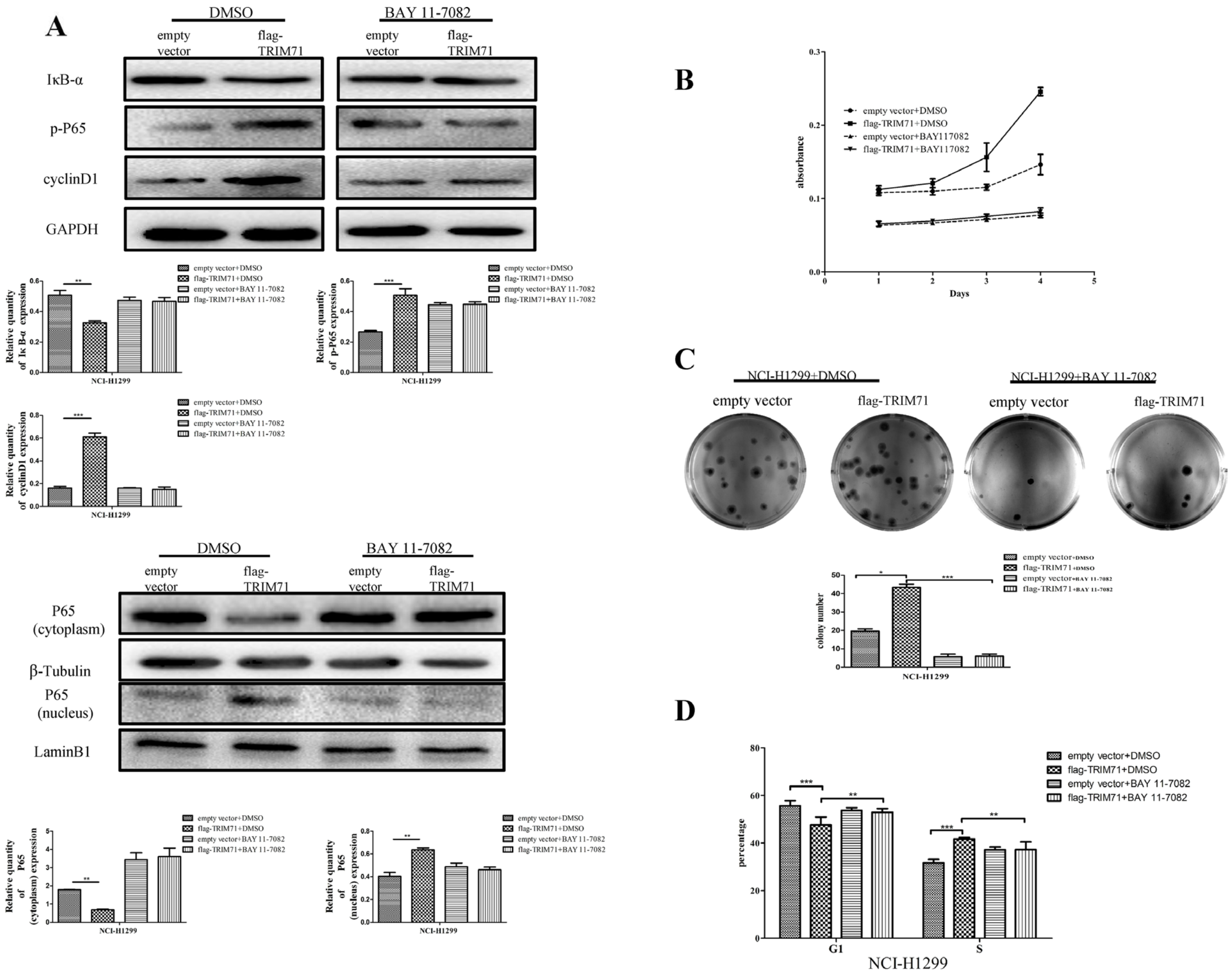

Supplementary Figure 3: TRIM71 regulates the proliferation of non-small cell lung cancer cells through the IκB-α/NF-κB pathway in NCI-H1299 cells. (**A**) Cells were treated with the NF-κB pathway inhibitor BAY 11-7082 or DMSO as a control, and the effects of TRIM71 expression on the expression of cyclin D1 and the phosphorylation of p65 were evaluated. Correlation between TRIM71 and these proteins was cancelled after using the NF-κB inhibitor BAY 11-7082. $^{**}p < 0.01$. (**B**) MTT assays were performed to evaluate the effects of TRIM71 expression and the NF-κB inhibitor BAY 11-7082 on cell proliferation. $^{*}p < 0.05$. (**C**) Colony formation assays were performed to evaluate the effects of TRIM71 expression and the NF-κB inhibitor BAY 11-7082 on colony formation. $^{*}p < 0.05$, $^{***}p < 0.001$. (**D**) Flow cytometry was performed to evaluate the effects of TRIM71 expression and the NF-κB inhibitor BAY 11-7082 on the G1/S phase transition. $^{**}p < 0.01$, $^{***}p < 0.001$.

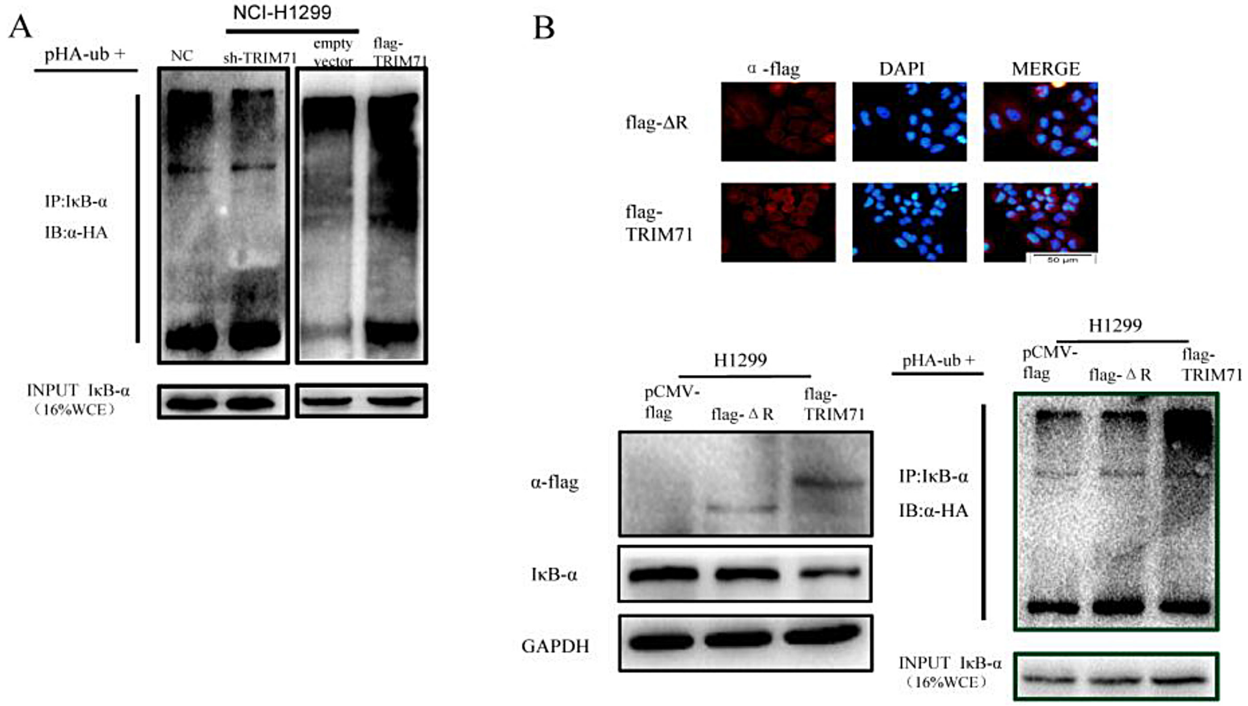

Supplementary Figure 4: TRIM71 is involved in IκB-α ubiquitination and degradation through its RING finger structure, affecting the NF-κB pathway in NCI-H1299 cells. (**A**) Effects of TRIM71 expression on the ubiquitination of IκB-α. Flag-tagged wild-type of TRIM71 was expressed in cells along with HA-ubiquitin (Ub). The levels of IκB-α ubiquitylation were evaluated by the immunoprecipitation of IκB-α using anti-IκB-α antibody followed by anti-HA immunoblotting. (**B**) Immunofluorescence staining showed the expression and localization of the transfected pcmv-flag TRIM71 plasmid with deletion of the RING domain and pcmv-flag TRIM71 plasmid. And immunoblotting was performed determine the effects of RING-deleted TRIM71 on IκB-α protein levels. Ubiquitination assays of IκB-α. Flag-tagged wild-type or RING finger domain-deleted mutants (ΔR) of TRIM71 were expressed in cells along with HA-ubiquitin (Ub). The levels of IκB-α ubiquitylation were evaluated by immunoprecipitation of IκB-α using anti-IκB-α antibodies followed by anti-HA immunoblotting. TRIM71 did not reduce the expression of IκB-α or increase the ubiquitination level of IκB-α after deletion of the RING domain.
